# Case report: Rare benign obstructive disease of the biliary tract—a rare case of eosinophilic cholangitis

**DOI:** 10.3389/fmed.2023.1340667

**Published:** 2024-01-17

**Authors:** Xiao-Ning Huang, Qi-Min Fang, Yu-Feng Zhong, Jing Liu

**Affiliations:** ^1^Department of Radiology, The Second Affiliated Hospital, Nanchang University, Nanchang, Jiangxi, China; ^2^Department of Pathology, Second Affiliated Hospital of Nanchang University, Nanchang, Jiangxi, China

**Keywords:** eosinophilic cholangitis, hypereosinophilic syndrome, bile duct, glucocorticoid, primary sclerosing cholangitis, primary biliary cholangitis

## Abstract

**Objective:**

Eosinophilic cholangitis (EC) is an uncommon form of benign biliary obstruction. It frequently eludes accurate clinical diagnosis, leading to inappropriate treatment methods. It is our aspiration that this clinical report will impart comprehensive insights into EC and, specifically, the critical role of tomographic examination.

**Case summary:**

A 34-year-old man was urgently admitted to the hospital due to excruciating abdominal distress persisting for several hours. Following a six-day course of anti-inflammatory therapy, his symptoms displayed marginal improvement, prompting his discharge. He returned to the hospital a month later for re-examination on doctor’s orders. Based on the results of the re-examination, the patient refused steroid hormone shock therapy and subsequently underwent laparoscopic left-lateral hepatic lobectomy in order to confirm the diagnosis. The preoperative absolute counts of eosinophils in the peripheral blood were documented as 2.3 × 10^9^/L, 3.06 × 10^9^/L, and 1.50 × 10^9^/L consecutively; concurrently, the corresponding percentages of eosinophils were quantified at levels of 21.90%, 30.70%, and 19.20%. The subsequent postoperative pathological assessment unveiled EC as the definitive diagnosis. The patient has since remained free from disease recurrence and is presently alive.

**Conclusion:**

When encountering a patient presenting with persistent elevation in absolute eosinophil count in peripheral blood, coupled with imaging manifestations suggestive of intrahepatic periductal inflammation, diagnosis of EC should be highly suspected. The most optimal diagnostic and therapeutic workflow for EC could entail CT-guided liver lesion biopsy, ensued by glucocorticoid pulse therapy, and finally, short-term monitoring utilizing CT or MRI (including T1WI, T2WI, DWI, CEMRI) techniques.

## Highlights

- Eosinophilic cholangitis (EC) is often misidentified as cholangiocarcinoma prior to surgery. Surgical resection is the common course of action for most patients.
- In cases where patients exhibit a persistent rise in absolute eosinophil count in peripheral blood, alongside imaging manifestations such as multisegmental annular thickening of the bile duct wall, accompanied by the characteristic rat-tail sign, beaded dilatation, and migratory sign during follow-up, EC should be considered as the likely diagnosis.
- CT-guided needle biopsy presents an alternative to choledochoscopy for achieving precise diagnostic results.
- Diagnostic glucocorticosteroid pulse therapy is the favored treatment option for EC.

## Introduction

Cholangitis refers to the inflammation of the bile duct system, often accompanied by symptoms such as right upper abdominal pain or discomfort below the xiphoid process, high fever, chills, jaundice (yellowing of the skin and sclera, dark urine), and other manifestations. Various factors can contribute to cholangitis, including biliary tract stones, tumors, bacterial or parasitic infections, biliary tract damage, and autoimmune diseases. Among these, biliary tract stones and bacterial infections are more commonly observed (1). Cholangitis and biliary obstruction exhibit a synergistic impact, necessitating differentiation from malignant biliary obstruction.

Eosinophilic cholangitis (EC) is a rare form of benign biliary obstruction characterized by eosinophil infiltration (2). The diagnosis of EC currently poses challenges, as there is no established clinical diagnostic criterion. Histological examination serves as the gold standard for diagnosis. EC shares similarities in imaging characteristics with cholangiocarcinoma, suppurative cholangitis, and sclerosing cholangitis. This case report aims to provide comprehensive information about EC, particularly highlighting tomographic data, in order to enhance our understanding of this condition.

## Case presentation

A 34-year-old male presented with episodic pain in the right upper abdomen that commenced before a week. The pain episodes lasted for 2–3 h each time, without an identifiable cause. The patient had not sought medical attention initially, but after 5 days, the abdominal pain became continuous and unbearable. He did not experience fever, chills, nausea, vomiting, or jaundice.

During the physical examination, mild tenderness was observed in the right upper abdomen, with no yellowing of the skin or sclera. There was no rebound tenderness, and Murphy’s sign was negative.

Laboratory tests revealed the levels of white blood cell count, the absolute eosinophil count (AEC)，the eosinophil percentage and the absolute monocyte count were elevated, respectively measured at 10.5 × 10^9^/L, 2.3 × 10^9^/L, 21.90%, and 0.79 × 10^9^/L. γ-glutamyltransferase was found to be elevated at 159.50 U/L, and the level of the tumor marker carbohydrate antigen (CA)125 was observed to be elevated, measured at 110.10 U/mL. Hepatitis B tests showed positive results for HBsAg, HBeAb, HBcAb, and Pre-S1. And other tests showed negative results for AMA, ANA, and IgG4.

**Figure 1:**
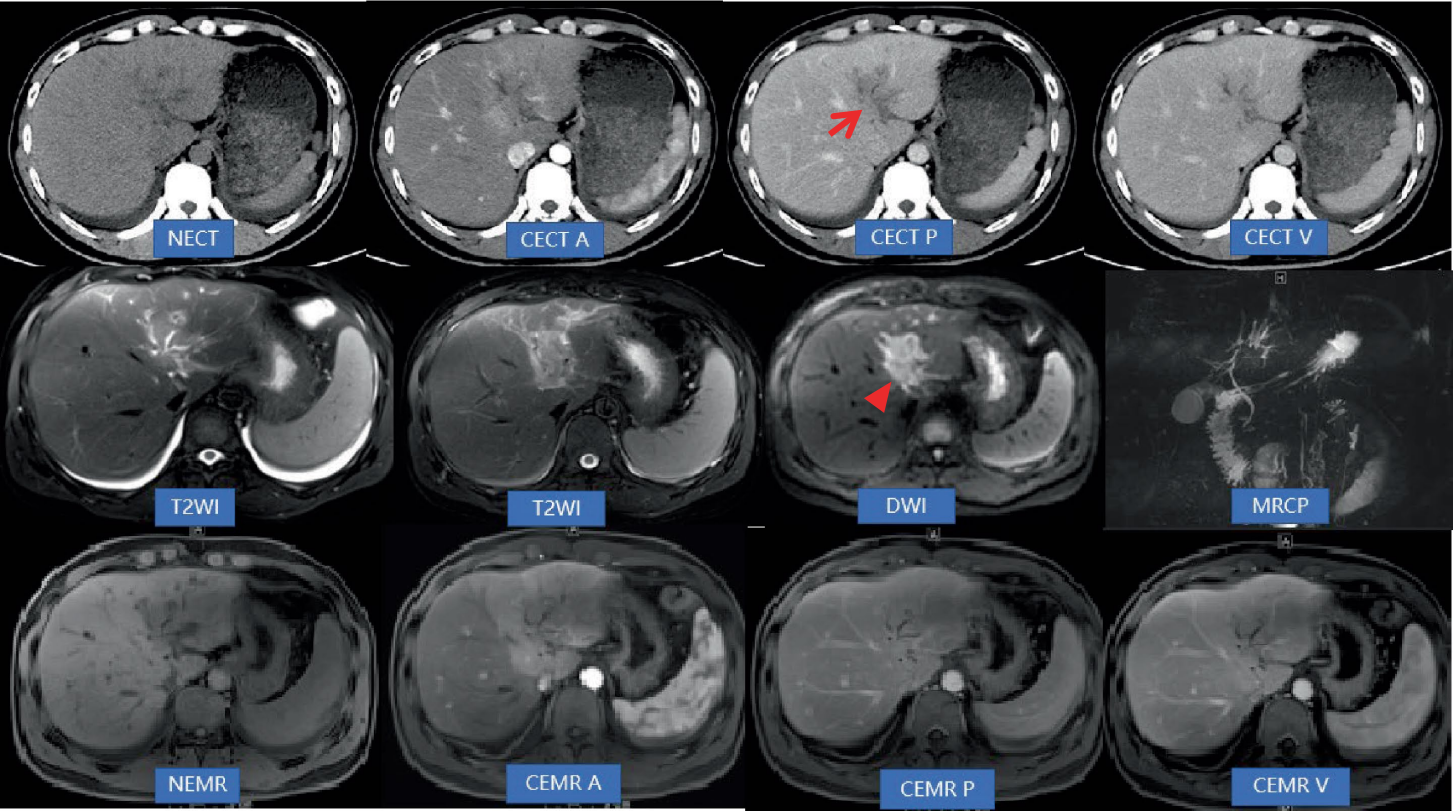
Abdominal non-contrast-enhanced computed tomography (NECT) and contrast-enhanced computed tomography (CECT; A: Artery phase, P: Portal vein phase, V: Vein phase) revealed mild dilatation of the bile ducts in the left lobe of the liver. Additionally, low-density areas (indicated by an arrow) were observed along the hepatic longitudinal fissure, extending toward the hepatic hilum. The boundary of these inflammatory areas appeared unclear, accompanied by narrowing and indistinctness of the adjacent left branch of the portal vein. Inflammatory exudation was also present in front of the colon. Moreover, there was thickening of the peritoneum and omentum. During CECT scans, the lesions exhibited gradual and significant enhancement, displaying a consistent and uniform enhancement pattern. However, the density of the lesions at each stage was lower compared to that of the liver parenchyma. Abdominal non-contrast-enhanced MRI (NEMR) and contrast-enhanced MRI (CEMR; A: Artery phase, P: Portal vein phase, V: Vein phase) demonstrated that the inflammatory areas in Segment 3, near the hepatic hilum, appeared as areas with low signals on T1-weighted imaging and high signals on T2-weighted imaging. On diffusion-weighted imaging (b = 800), limited diffusion was observed within the lesions (indicated by a triangle). These lesions measured approximately 3.6 cm × 3.8 cm and displayed a crab-like edge, distinctly separate from the hilar bile duct and blood vessels. Regarding contrast enhancement, the lesions exhibited moderate progressive enhancement on CEMR. Furthermore, lymph nodes in the hilar region were found to have distinct and uniform enhancement on CEMR.

Computed tomography (CT) and magnetic resonance imaging (MRI) of the upper abdomen revealed the presence of multiple lesions in the longitudinal fissure of the liver, hepatic hilum and omentum (Figure 1).

After a multidisciplinary team discussion at our hospital, the patient was diagnosed with biliary tract obstruction caused by bile duct inflammation.

**Figure 2:**
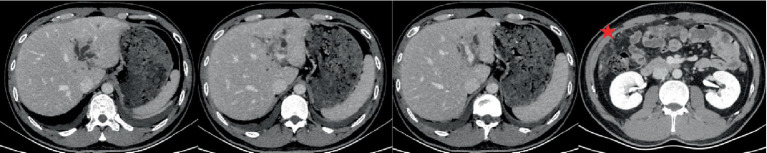
3D-computed tomography angiography provided a visual depiction of the lesion in the left lobe of the liver, showcasing its extension from the longitudinal fissure toward the patchy shadow in the hilar area. Furthermore, infiltration around the sagittal portion of the bile duct and portal vein was observed. There was notable exacerbation of bile duct dilatation and exudation in front of the colon (indicated by a pentagonal shape), which was more severe compared to previous imaging. Additionally, there was a significant and persistent increase in peripheral absolute eosinophil count.

In consideration of bile duct inflammation, the patient received cefotaxime sodium (1 g, twice daily, intravenous drip), entecavir capsules (0.5 mg, once daily, orally), and ornidazole sodium chloride injection (0.5 g/100 mL, every 12 h, intravenous drip) for antiviral and anti-inflammatory treatment. After 6 days of anti-inflammatory treatment, there was a slight improvement in right upper abdominal distension and pain. Follow-up blood tests showed elevated white blood cell count, AEC, eosinophil percentage, and absolute monocyte count, respectively measured at 9.96 × 10^9^/L, 3.06 × 10^9^/L, 30.70%, and 0.73 × 10^9^/L. The level of C-reactive protein was found to be elevated, measured at 24.51 mg/L. The inflammatory areas showed enlargement, and intrahepatic bile duct dilation worsened (Figure 2).

**Figure 3:**
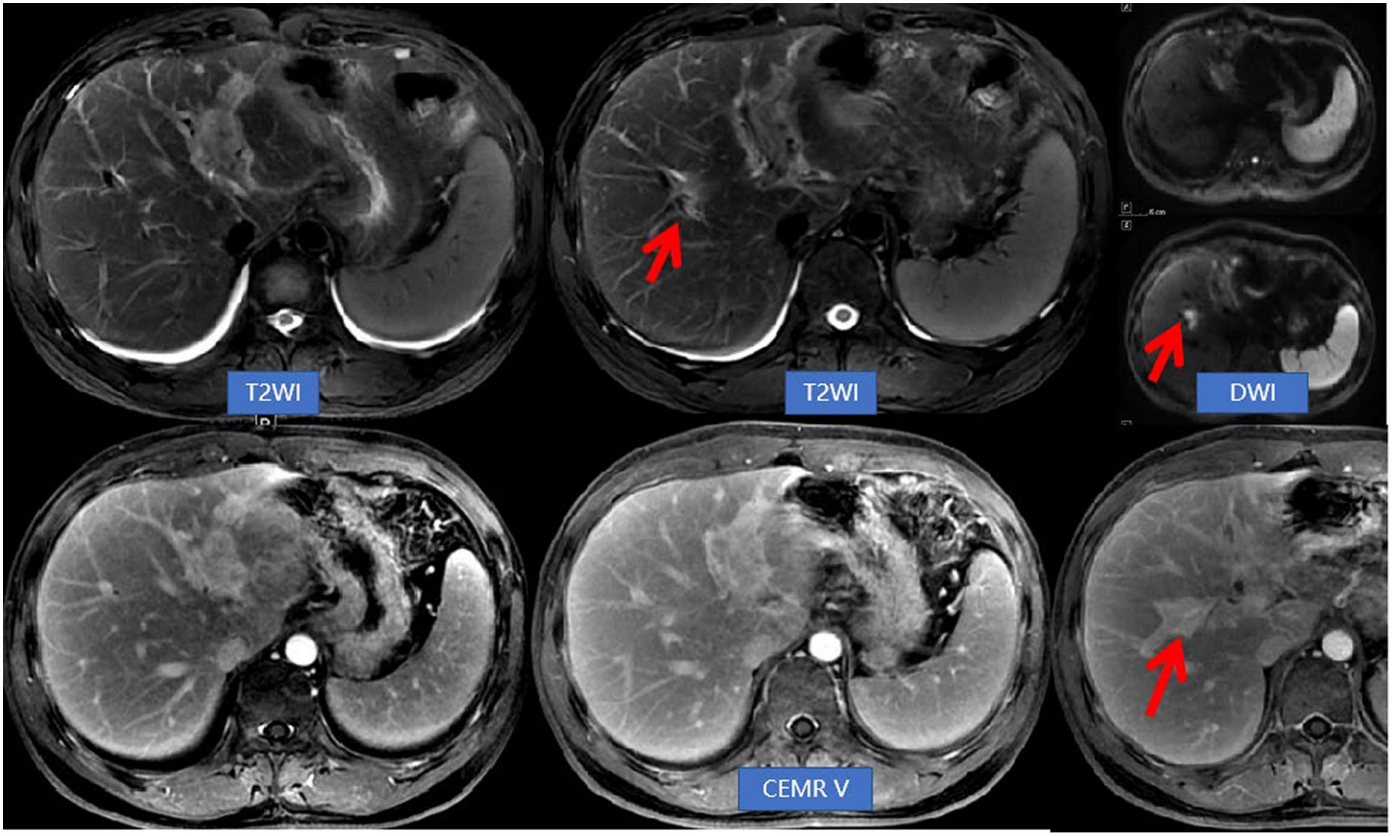
Abdominal NEMR and CEMR revealed a reduction in lesions within the left hepatic lobe and hepatic hilum compared to previous findings. Additionally, there was an improvement in bile duct dilatation, while a new lesion (indicated by arrows) near the right hepatic duct was observed. This imaging feature was referred to as migratory sign.

**Figure 4:**
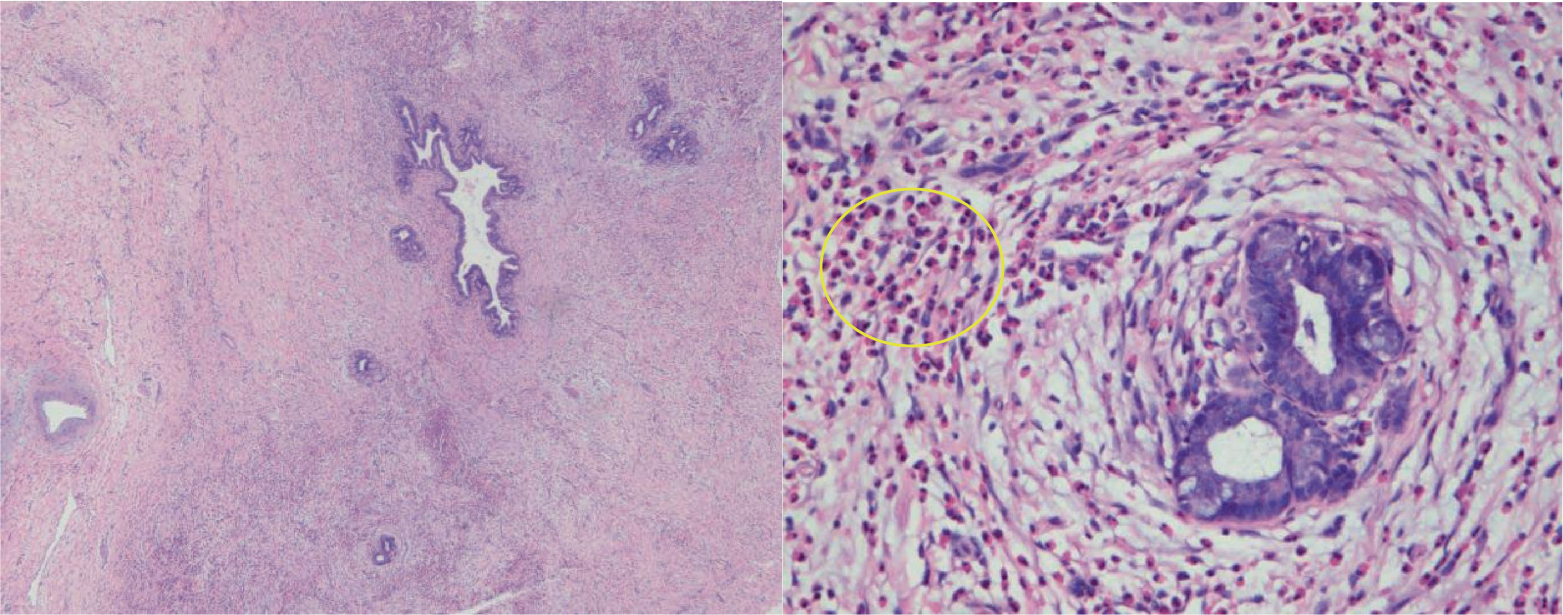
Pathological examination (HE staining, 4× and 40× magnification) revealed a uniform and significant thickening of the bile duct walls accompanied by eosinophil infiltration. Additionally, there was a notable presence of eosinophilic infiltration (indicated by a ring) within the adjacent adipose tissue space.

The patient was discharged from the hospital after experiencing an improvement in symptoms. A month later, he returned for routine blood tests and repeat MRI examination of the upper abdomen. The eosinophil percentage was found to be elevated, measured at 19.20%. While the original biliary duct lesions had decreased in size, new lesions with a migratory sign were observed on the upper abdominal MRI (Figure 3). The medical team suggested diagnostic glucocorticosteroid pulse therapy or needle biopsy, but the patient declined these options and instead opted for a laparoscopic left-lateral hepatic lobectomy. The postoperative pathological examination confirmed the diagnosis of EC (Figure 4). The AEC level was measured at 0.28 × 10^9^/L 1 month post-operation and subsequently decreased to 0.09 × 10^9^/L after 1 year.

## Discussion

Hypereosinophilic syndrome (HES) is a specific type of idiopathic eosinophilia that is differentiated from congenital, secondary, and clonal eosinophilia (3). The diagnosis of HES requires the presence of ongoing eosinophilia (absolute eosinophil count ≥1,500 cells/μL for at least 6 months) along with organ damage, such as the skin, heart and major blood vessels, lungs, gastrointestinal tract, hepatobiliary pancreas, or nervous tissue. Other known causes of eosinophilia, such as parasitic infections, collagen vascular diseases, and allergies, must be ruled out (4). HES involving the digestive system is uncommon, with fewer than 100 cases reported globally. Involvement of the bile duct is a rare occurrence in HES. Eosinophilic cholangitis (EC) is characterized by persistent elevation of eosinophils in the peripheral blood, infiltration of the bile duct wall leading to thickening, fibrosis resulting in bile duct obstruction, and hepatic dysfunction.

Clinical manifestations of EC include right upper abdominal pain, jaundice, and low-grade fever. Laboratory tests reveal elevated absolute eosinophil count (AEC) and eosinophil percentage, as well as increased liver enzymes and bilirubin levels. CT imaging shows uniform thickening of the walls of both intrahepatic and extrahepatic bile ducts, resulting in luminal stenosis represented by a “rat-tail sign,” accompanied by dilation of the bile duct upstream. EC may extend into the hilar, perihepatic, or peritoneal fat spaces (5–9). Currently, cholangioscopic histopathological biopsy is considered the gold standard for diagnosing EC. However, in many cases, EC is misdiagnosed as cholangiocarcinoma preoperatively, leading to surgical resection. The primary treatment for EC is corticosteroids (preferably glucocorticoids like prednisone), and if necessary, hydroxyurea and interferon-α can be used as second-line medications. In cases of refractory hypereosinophilic syndrome (HES), an interleukin-5 antagonist monoclonal antibody called mepolizumab can be considered (10). Surgical resection may be considered as a follow-up treatment after initial drug therapy for EC.

We compared this case with published case reports on EC and summarized the findings in two tables. Table 1 includes the clinical manifestations of EC patients, while Table 2 covers the diagnosis and treatment approaches for EC patients. The analysis of the tables revealed that the gender and age of onset in this case were consistent with the literature. However, there were some differences in the clinical symptoms. The patient experienced acute right upper abdominal pain lasting 2–3 h each time, which slightly differed from the reported cases. Additionally, the patient’s peripheral blood eosinophils gradually increased, peaking at 30.7%, with significant short-term fluctuations. Abnormal liver enzyme indexes, the negative results for AMA, ANA and IgG4 were in line with previous literature. However, this case showed elevated CA12-5, which differs from what is reported and might be due to retinal exudation. The imaging findings in this case also diverged from the literature in several aspects: (1) CT re-examination within 1 week indicated disease progression; (2) MRCP revealed rat tail stenosis and beaded dilatation of the intrahepatic bile duct; (3) follow-up on MRI a month later demonstrated migratory sign of the lesions. Considering these imaging findings, the progressive elevation of peripheral blood eosinophils, and slight symptom relief following anti-inflammatory therapy, the MDT discussion suggested a probable diagnosis of EC. Therefore, further invasive examinations, such as ERCP, choledochoscopy and Endosonography, were not performed. However, CT-guided liver lesion biopsy is at risk of bleeding because the lesion surrounds the portal vein and hepatic artery. Eventually, the patient declined diagnostic steroid therapy due to concerns about side effects, opted for laparoscopic left lobectomy due to the fear of a malignant tumor. The patient recovered uneventfully without recurrence.

**Table 1:** Clinical manifestations of EC patients.

Cases	Authors	Sex	Age	Course of disease	Symptoms	Signs	Laboratory tests						
							Peak eosinophils percentage (%)	Total bilirubin (mg/dL)	AST (U/L)	ALT (U/L)	AMA	ANA	IgG4
1	Matsumoto et al. (8)	F	38	3 days	Epigastralgia and back pain	Jaundice, epigastric tenderness	15.3	6.63	151	400	/	Negative	Negative
2	Nashed et al. (2)	M	33	2 months	Fatigue, jaundice, Severe pruritis, and steatorrhea	Scleral icterus, mild tenderness on deep palpation in the right upper quadrant	2	5.2	75	208	Negative	Negative	Negative
3	Dodda et al. (5)	F	71	2 weeks	Fatigue, abdominal pain	Negative	27.2	0.3	193	302	Negative	Negative	Negative
4	Hirata et al. (6)	M	67	Postoperative follow-up of rectal cancer	Negative	Negative	Normal	Normal	Normal	Normal	/	/	/
5	Fukatsu et al. (9)	M	15	1 month	Itching, jaundice, grayish white stool, and abdominal pain	Jaundice	9.6 (Approximate)	22.6	83	75	Negative	Negative	Negative
6	Our case	M	34	a week	Episodic pain in the right upper abdomen	Mild tenderness	30.7	12.1	30.6	51.9	Negative	Negative	Negative

AST, Aspartate aminotransferase; ALT, Alanine aminotransferase; AMA, Aspartate aminotransferase; ANA, Antinuclear antibodies; /, undetected; %eosinophils (ULN < 5%).

**Table 2:** Diagnosis and treatment of EC patients.

Cases	Authors	Noninvasive examinations			Invasive examinations			Diagnosis	Treatment	Outcome
		US	CT	MRI + MRCP	ERCP	Choledochoscope	Endosonography			
1	Matsumoto et al. (8)	Isoechoic solid lesion in the common hepatic duct, thickened wall of intrahepatic bile ducts with well enhanced	Intrahepatic bile duct dilation, a extrahepatic bile duct solid lesion with slightly enhanced	/	Severe narrowing from common hepatic duct to porta hepatis with dilation of intrahepatic bile ducts.	/	A solid, isoechoic lesion around the common hepatic, duct.	EC	Prednisolone, 60 mg/d intravenously, which was reduced gradually by 10 mg per week.	Recovery
2	Nashed et al. (2)	Markedly dilated intrahepatic and extrahepatic bile ducts	The intrahepatic bile duct and CHD were obviously dilated	Centripetal, rat-tailed stenosis at the beginning of the CBD, The CBD and pancreatic duct were normal in caliber	To dilate the narrow biliary tract and perform endoprostheis	Confirmed a CHD stricture, biopsy failed to produce a definitive pathological diagnosis	A mass in the proximal CHD with wall thickness of 4.7 mm	/	Exploratory laparotomy, an resection of the entire CBD	Recovery
3	Dodda et al. (5)	Abnormal thickening of the CBD, pericholecystic fluid, and gallbladder sludge without wall thickening	/	The mid CHD was focal narrowing with upstream biliary dilation, tapering at the ampulla, and no filling defects	CHD narrowing and a distal CBD stricture with extra and intrahepatic dilation	Frond-like ductal lesions in the middle CBD	Extrahepatic biliary dilation, irregular and diffusely thickened bile duct walls, and no focal mass	EC	As the patient’s symptoms improved, no further treatment was given, a follow-up strategy was adopted	Recovery
4	Hirata et al. (6)	/	A nodules in the proximal bile duct	/	The filling defects in the proximal bile duct	The whitish nodules with neither irregular vessels nor atypical mucosa on the surface except for a simple dilated vessel	/	EC	No treatment	/
5	Fukatsu et al. (9)	/	Dilatation and wall thickness of the intrahepatic bile ducts	A stricture of the intrahepatic and extrahepatic bile duct	A stricture of the left and right intrahepatic and extrahepatic bile duct	/	/	EC	UDCA	Recovery
6	Our case	Mild dilation of the intrahepatic bile duct without definitive signs of lithiasis or tumors	Multiple lesions in liver, hepatic hilum and omentum, the intrahepatic bile duct dilation, progression of the lesion within a week	High signals on T2WI, high signals on DWI, intrahepatic bile duct rat-tail stenosis, beaded dilatation, migratory sign during follow-up	/	/	/	EC probable	Laparoscopic left-lateral hepatic lobectomy	Recovery

MRCP, Magnetic Resonance Cholangiopancreatography; ERCP, Endoscopic Retrograde Cholangiopancreatography; CHD, Common Hepatic Duct; CBD, Common Bile Duct; /, Undetected or NA (not available); UDCA, Ursodeoxycholic Acid; T2WI, T2-weighted Imaging; DWI, Diffusion-weighted Imaging.

EC is a rare cause of non-malignant biliary obstruction that needs to be distinguished from obstructions caused by malignant tumors. Cholangiocarcinoma, the most common type of malignant biliary obstruction, is more frequently found in middle-aged and older adults, often occurring in the left lobe of the liver. Elevated bilirubin levels and the tumor marker CA19-9 are typically observed. Imaging examinations including B-ultrasound, CT, and MRI show dilatation of the intrahepatic bile ducts, stenosis of the hilar bile ducts, and an irregularly shaped, poorly defined soft tissue mass without a capsule. The bile duct walls may display heterogeneous thickening, and progressive enhancement of the tumor can be observed. Another cause of malignant biliary obstruction is a biliary tumor thrombus originating from primary hepatocellular carcinoma. This condition often occurs in patients with liver diseases such as viral hepatitis, alcoholic hepatitis, or fatty liver disease. Elevated alpha-fetoprotein or abnormal prothrombin (PIVKA-II) levels are indicative. The mass exhibits expansive or infiltrative growth, with a spherical or irregular shape. Liquefaction necrosis or hemorrhagic cystic changes are often present, and the tumor can invade the bile duct wall, forming an embolus that obstructs the bile duct. The obstruction primarily affects limited segments of the biliary tract, appearing as intraluminal nodules or columnar soft tissue shadows, with a washout-type enhancement pattern observed during imaging.

EC should be differentiated from Primary Sclerosing Cholangitis (PSC), Primary biliary cholangitis (PBC), IgG4 sclerosing cholangitis and recurrent suppurative cholangitis as other causes of inflammatory areas in benign bile duct obstruction. Primary sclerosing cholangitis (PSC) is a rare cholangiopathy characterized by multifocal bile duct stenosis and progressive liver disease. This disease is more common in males and has a bimodal distribution of age of onset, with peaks in 15- and 35-year-olds. The clinical manifestations of PSC vary, with early-stage symptoms such as asymptomatic status, right upper abdominal pain, fatigue, jaundice, pruritus, and fever, gradually progressing to late-stage cirrhosis-related symptoms. Some patients are diagnosed with PSC following medical consultation for inflammatory bowel disease (IBD). Laboratory tests usually show no specific indicators. The diagnosis of PSC primarily relies on bile duct imaging and liver histopathology. Imaging findings include multifocal and short-segmental wall thickening of the bile ducts within and outside the liver, wall rigidity, annular stenosis of the lumen, expansion of the bile ducts in the upper portions of stenosis, presenting a beaded appearance, and dry tree-like changes in the bile ducts with disease progression. Primary biliary cholangitis (PBC) is a chronic autoimmune intrahepatic cholestasis disorder that primarily affects women, typically in middle-aged and elderly individuals. The most common clinical presentations include fatigue and pruritus, while laboratory findings reveal elevated alkaline phosphatase (ALP) and gamma-glutamyl transpeptidase (GGT) levels. Immunologically, the disease is characterized by positive anti-mitochondrial antibodies (AMAs), positive ANA, and increased serum immunoglobulin M (IgM). Radiological imaging examinations often appear normal during the early stages of the disease, with no evidence of biliary dilatation or obstruction. As the disease progresses, imaging findings may reveal cirrhosis and portal hypertension. MRCP is helpful in differentiating PBC from bold tubular PSC. Extrahepatic manifestations of PBC often occur in conjunction with other immune-mediated conditions such as Sjogren’s syndrome, systemic lupus erythematosus, inflammatory bowel disease, and others. IgG4 sclerosing cholangitis can manifest as a form of multisystemic disease within IgG4-related disease or solely affect the bile ducts. Laboratory examinations reveal a significant increase in serum IgG4 levels, as well as elevated blood cell sedimentation rate and C-reactive protein. Imaging of IgG4 sclerosing cholangitis demonstrates diffuse involvement of the intrahepatic and extrahepatic bile ducts, with annular homogeneous wall thickening. The lumen exhibits rat tail-like stenosis and beaded expansion, with notable and delayed enhancement. Recurrent suppurative cholangitis, on the other hand, is characterized by recurrent episodes of cholangitis and the presence of bile duct stones. It is often accompanied by atrophy of the affected hepatic segment and clinically presents with recurrent fever and jaundice. Imaging shows mild diffuse thickening of the long segmental duct wall with marked enhancement. Luminal stenosis is not prominent and is frequently associated with gas accumulation within the biliary tract and the presence of calculi.

Referring to the clinical guidelines and literature on cholangitis, this paper compares the examination methods used in the management of several cases presented herein, aiming to facilitate a more rational selection of examination techniques for such patients. Among the 6 EC patients, the frequencies of non-invasive examinations using US, CT, and MRI were 2/3, 5/6, and 2/3, respectively, while the frequencies of invasive examinations involving ERCP, choledochoscope, and endoscopy were 5/6, 1/2, and 1/2, respectively. Prior to the diagnosis of EC patients, CT and ERCP were the most frequently employed techniques, possibly due to their early adoption and high technical proficiency. Preoperative CTA examination allows for accurate assessment of biliary duct dilation, demonstrates the bile duct wall and extramural lesions, and displays the three-dimensional anatomical relationships between the biliary ducts and the hepatic artery, portal vein, and hepatic vein. This information can guide clinicians in developing preoperative plans to reduce or avoid surgical complications. Compared to CT, US, and MRI, which lack radiation advantages, these imaging modalities are valuable for detecting lesions and managing the disease course of EC patients (11, 12). In particular, MRCP serves as a gold standard for diagnosing and preoperatively evaluating biliary tract diseases due to its non-invasive characteristic, convenience, and high repeatability (13, 14). In this case, a US examination revealed mild bile duct dilation, absence of calculi and tumor signs, but no inflammatory exudation surrounding the bile duct. This finding may be associated with the operator’s technical skills and diagnostic abilities. Choledochoscopic tissue biopsy currently serves as the diagnostic gold standard for EC. However, due to the challenges associated with its implementation, it has not been widely adopted and faces difficulties in achieving broader usage. As an alternative, CT-guided needle biopsy can be employed for diagnosing EC. Based on the diagnosis and treatment approach utilized in this case, the management of EC can be divided into three steps: CT-guided puncture biopsy, diagnostic glucocorticosteroid pulse therapy, and short-term follow-up CT or MRI examination. This diagnostic and therapeutic approach offers advantages such as reduced invasiveness, ease of manipulation, and potential for widespread application.

## Conclusion

In patients presenting with right upper abdominal pain and distension, along with persistently elevated peripheral blood AEC, imaging findings that include wall thickening of multiple segments of the bile duct with rat tail-like stenosis and beaded dilatation, notable enhancement of lesions, inflammatory exudation in the surrounding tissue, and migratory sign during follow-up, a probable diagnosis of EC should be considered. CT and MRI, especially MRCP, play an important role in the diagnosis of EC. Ultrasound and MRI can play a role in the follow-up of EC. The three types of biopsies mentioned (choledochoscopic biopsy, endoscopic ultrasound biopsy, and CT-guided biopsy) can all be used to extract diseased tissue for diagnosis, thereby avoiding surgical resection. CT-guided tissue biopsy is relatively easier to perform than choledochoscopic biopsy and endoscopic ultrasound biopsy. For EC patients with impaired function of target organs, steroid hormone therapy can be used, while EC patients without clinical symptoms and abnormal target organs can be followed up without intervention.

## Data availability statement

The original contributions presented in the study are included in the article/supplementary material, further inquiries can be directed to the corresponding author.

## Ethics statement

Written informed consent was obtained from the individual(s) for the publication of any potentially identifiable images or data included in this article.

## Author contributions

X-NH: Writing – original draft, Writing – review & editing, Project administration. Q-MF: Writing – review & editing. Y-FZ: Data curation, Resources, Writing – review & editing. JL: Data curation, Writing – original draft.
